# Supplementary material for: Differences in HIV-1 reservoir size, landscape characteristics and decay dynamics in acute and chronic treated HIV-1 Clade C infection
Source: medRxiv. 2024 Jun 10:2024.02.16.24302713. Preprint. [Version 2] doi: 10.1101/2024.02.16.24302713 (PMC11213047; doi:10.1101/2024.02.16.24302713)
Supplement: Supplementary file 1 [file NIHPP2024.02.16.24302713v2-supplement-1.pdf]

Supplementary Table 1: Clinical and biological characteristics of 35 study participants

Identifier	HLA-A1	HLA-A2	HLA-B1	HLA-B2	HLA-C1	HLA-C2	Fiebig Stage at Detection	Timing of Treatment Initiation (DPOPV)	Peak Viral Load	ddPCR	FLIP Seq
**FRESH_002**	02:01	30:01	44:03	**58:02***	04:01	06:02	I	724	5,70E+07	✓	✓
**FRESH_003**	02:01	30:01	15:10	42:02	08:04	17	I	310	8,20E+06	✓	✓
**FRESH_005**	24:02	29:02	07:02	44:03	07:01	07:02	I	809	5,20E+07	✓	✓
**FRESH_004**	03:01	**74:01****	15:03	**58:02**	02:10	06:02	I	346	3,00E+07	✓	✓
**FRESH_010**	23:01	30:01	15:10	**58:01**	03:02	16:01	III	668	5,70E+07	✓	✓
**FRESH_011**	23:01	68:02	08:01	**58:01**	03:04	07:01	I	427	1,10E+07	✓	✓
**FRESH_007**	23:01	**74:01**	35:01	**58:01**	04:01	06:02	I	456	4,00E+06	✓	✓
**FRESH_013**	01:01	66:01	39:10	**81:01**	12:03	18	I	1202	7,70E+05	✓	✓
**FRESH_009**	43:01	43:01	07:02	15:03	02:10	18	I	416	9,90E+06	✓	✓
**FRESH_008**	02:05	02:05	**58:01**	**58:01**	07:01	07:01	I	297	9,50E+06	✓	✓
**FRESH_012**	23:01	29:02	42:01	53:01	03:04	17	I	1059	1,20E+07	✓	✓
**FRESH_015**	30:02	34:02	08:01	44:03	04:01	07:01	I	1	2,30E+04	✓	✗
**FRESH_018**	68:02	**74:01**	15:03	**57:02**	02:10	18	V	2	1,50E+02	✓	✓
**FRESH_016**	01:23	30:02	42:01	**58:01**	06:02	17	I	1	3,40E+04	✓	✓
**FRESH_029**	30:01	30:01	39:10	42:02	12:03	17:01	I	1	1,40E+04	✓	✗
**FRESH_019**	02:01	03:01	**58:01**	**58:02**	03:02	06:02	I	1	3,60E+05	✓	✓
**FRESH_033**	68:02	68:02	07:02	15:10	03:04	07:02	I	1	4,40E+02	✓	✓
**FRESH_022**	30:01	30:02	**18:01**	53:01	04:01	07:04	I	1	2,10E+05	✓	✓
**FRESH_025**	01:01	30:01	42:01	47:01	06:02	17	I	3	8,90E+04	✓	✓
**FRESH_024**	01:01	29:01	44:03	**81:01**	08:04	18	I	1	5,90E+03	✓	✓
**FRESH_030**	02:05	29:02	42:01	**58:01**	07:01	17:01	III	1	2,00E+07	✓	✗
**FRESH_023**	02:05	23:01	08:01	14:01	07:01	08:04	I	3	4,10E+04	✓	✓
**FRESH_036**	02:05	68:01	35:01	**58:01**	04:01	07:01	I	1	1,90E+04	✓	✗
**FRESH_031**	01:01	68:02	14:01	**81:01**	08:02	18	I	1	1,70E+04	✓	✓
**FRESH_038**	30:01	30:02	08:01	15:03	02:10	07:01	I	1	4,60E+02	✓	✗
**FRESH_028**	03:01	29:02	44:03	**58:02**	06:02	07:01	I	2	2,60E+06	✓	✓
**FRESH_035**	29:02	33:03	07:02	44:03	07:01	07:02	I	1	7,70E+02	✓	✗
**FRESH_032**	30:01	**74:01**	15:03	**18:01**	02:10	07:04	I	1	7,30E+03	✓	✓
**FRESH_034**	30:02	33:03	53:01	**58:02**	04:01	06:02	I	2	8,60E+04	✓	✓
**FRESH_039**	02:01	02:05	08:01	**58:02**	06:02	07:01	I	1	2,50E+03	✓	✗
**FRESH_048**	30:01	30:02	15:03	**57:03**	02:10	07:01	I	1	1,50E+04	✓	✓
**FRESH_041**	29:02	74:01	44:03	**57:03**	07:01	07:01	I	1	1,40E+03	✓	✗
**FRESH_046**	23:01	30:01	15:10	18:01	07:04	16:01	I	1	2,50E+03	✓	✗
**FRESH_042**	29:02	30:02	07:02	44:03	07:01	07:02	III	1	1,50E+04	✓	✗
**FRESH_044**	01:01	02:05	42:02	**81:01**	17:01	18	I	1	6,50E+04	✓	✗

*Deleterious HLA class I alleles (red), **protective HLA class I alleles (green).

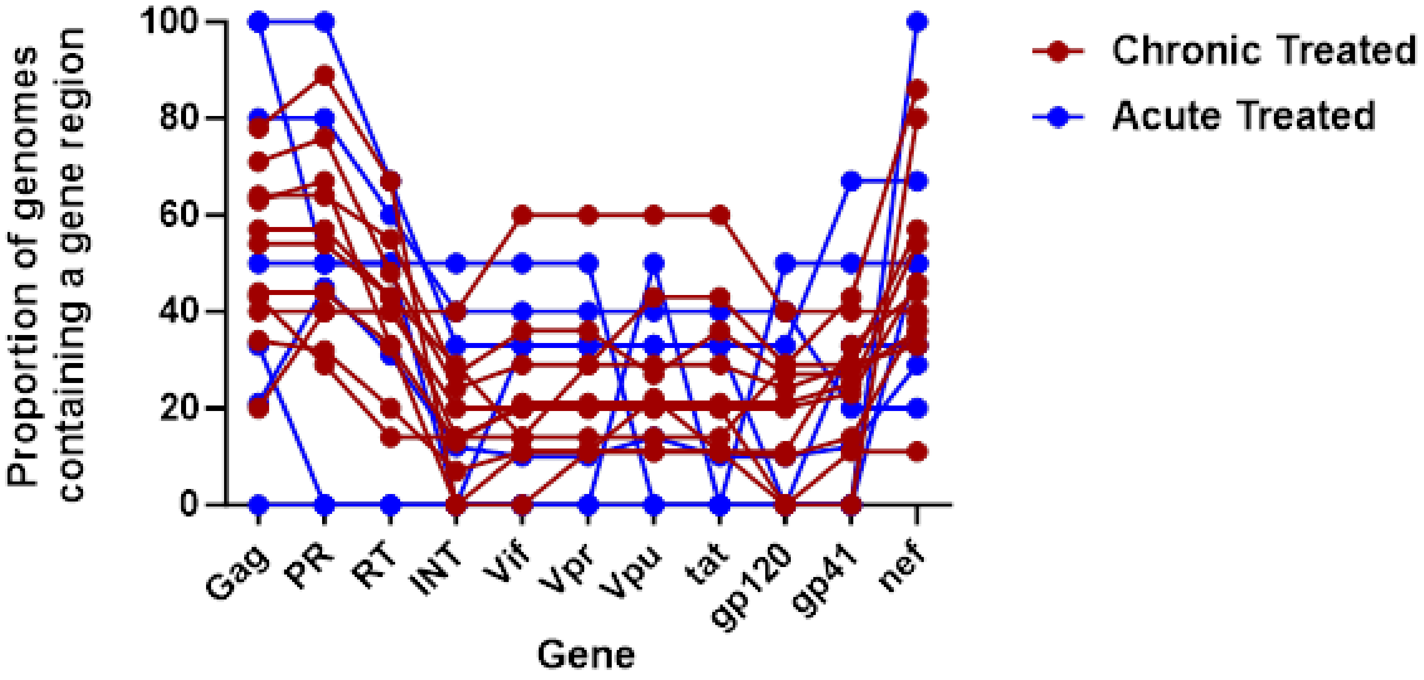

Supplementary Figure 1: In this cohort of HIV-1 subtype C, genome deletions were most frequently observed between between *integrase* and *envelope* relative to Gag ($p<0.0001$–0.001).

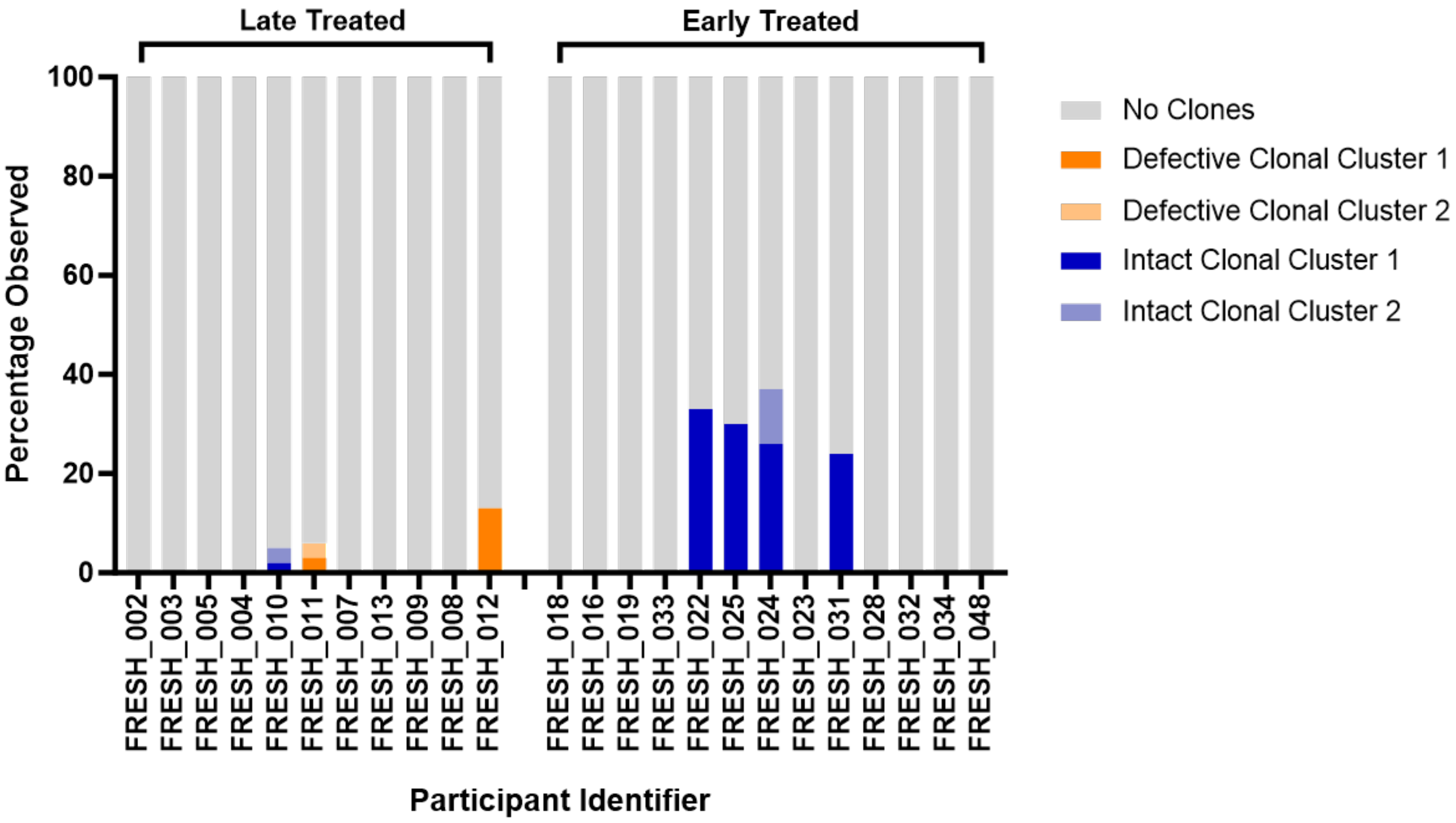

Supplementary Figure 2: Clonal expansion of infected cells was detected in both defective (orange) and intact (blue) genomes in late and early treated study participants. This analysis was performed with all sequences available for each participant at all time points.
